# Editorial for the Special Issue on Advanced Micro- and Nano-Manufacturing Technologies, 2nd Edition

**DOI:** 10.3390/mi17080930

**Published:** 2026-08-04

**Authors:** Kun Li

**Affiliations:** 1 College of Mechanical and Vehicle Engineering, Chongqing University, Chongqing 400044, China; kun.li@cqu.edu.cn; 2 Chongqing Key Laboratory of High-Performance Structural Additive Manufacturing, Chongqing 400044, China; 3 State Key Laboratory of Mechanical Transmission, Chongqing University, Chongqing 400044, China

In recent years, high-energy-beam technologies and nano- and micro-scale advanced manufacturing have developed rapidly, injecting new vitality into modern engineering and expanding the possibilities for practical application. At the same time, these advances have raised higher requirements for both material systems and manufacturing approaches. For many years, one of the central challenges has been not only to develop advanced materials, but also to establish suitable processing methods that enable their full potential to be realized across scientific, technological, and industrial applications. In this evolving context, advanced manufacturing is no longer confined to the traditional notion of material shaping. Rather, it has grown into a broader and more integrated field that encompasses process innovation, structural and functional integration, precision fabrication, and intelligent production systems [1,2,3,4,5,6,7].

To fully leverage the benefits of advanced materials and components, significant research has been focused on advanced processes, novel manufacturing methodologies, post-processing technologies, and auxiliary numerical simulation and analysis. These aspects play a decisive role in determining the final quality of mechanical components, including dimensional accuracy, surface integrity, residual stress distribution, fatigue performance, wear resistance, and overall service reliability [2,3,8,9]. Particularly for components produced through high-energy or additive manufacturing processes, the as-built material often cannot meet engineering specifications without subsequent post-processing operations such as heat treatment, machining, polishing, and surface modification [8,9,10].

Meanwhile, modeling and numerical analysis have become indispensable tools for understanding the fundamental mechanisms of advanced manufacturing processes and for optimizing processing parameters. Multiphysics simulations are widely used to explore powder transport, melt-pool dynamics, heat and mass transfer, stress evolution, microstructure development, and material removal behavior in laser processing, additive manufacturing, and micro-/nano-fabrication [3,4,11,12,13]. These methods are particularly valuable in advanced manufacturing since many critical phenomena occur in highly transient and localized environments that are difficult to observe directly. Thus, simulation-assisted process analysis has become an effective strategy for reducing trial-and-error experimentation, improving process reliability, and accelerating technological development [3,4,14,15,16].

In parallel, advanced detection, monitoring, and intelligent control systems are reshaping modern manufacturing processes. Tool condition monitoring, in-situ sensing, defect detection, machine learning, and physics-informed intelligent control systems are enabling the transition from empirical parameter adjustment to closed-loop, data-driven, and self-optimized operation [17,18,19,20,21,22,23,24]. These developments not only enhance manufacturing consistency and product quality but also facilitate the realization of high-performance and functional manufacturing technologies on a global industrial scale [18,21,22,23].

This Special Issue, Advanced Micro- and Nano-Manufacturing Technologies, brings together 12 original research articles and one review paper, encompassing the following major themes: (1) high-energy, nano-, and micro-scale advanced manufacturing processes; (2) post-processing technologies for mechanical parts; (3) functional and graded materials enabled by advanced manufacturing methodologies; (4) modeling and numerical analyses in advanced manufacturing processes; and (5) advanced detection, monitoring, and intelligent control for manufacturing. Collectively, these contributions not only deepen our understanding of state-of-the-art manufacturing technologies, but also provide a solid scientific and technological foundation for the future advancement of the manufacturing industry. The key findings of these studies and their scientific significance are summarized below.

1. High-energy/nano/micro advanced manufacturing processes: Li et al. [Contribution 1] combined computational fluid dynamics (CFD) simulations with non-contact fixed-point polishing experiments to systematically investigate the relationship between flow-field evolution and material removal behavior in metal ultrasonic vibration polishing. Their study showed that cavitation significantly regulates pressure distribution, flow velocity, and surface damage morphology, thereby clarifying a synergistic material removal mechanism involving cavitation impact, mechanical scratching, and fatigue spallation. Chen et al. [Contribution 2] established a numerical model of TIG welding arcs under the influence of a longitudinal magnetic field and analyzed the effects of magnetic-field strength on arc shape, temperature field, pressure field, plasma velocity, and potential distribution. Their results demonstrated that the magnetic field can markedly alter arc constriction behavior and the coupling characteristics of multiple physical fields, providing new theoretical support for welding process control. As the review paper included in this Special Issue, Peng et al. [Contribution 3] systematically summarized recent progress in gas–solid two-phase flow technology for laser cladding, with particular emphasis on powder transport behavior, nozzle design, and powder-field uniformity control, and further discussed future development trends in this area. Taken together, these contributions highlight continuing advances in both high-energy-beam and micro-scale manufacturing, spanning fundamental mechanisms, process regulation, and critical reviews.
2. Post-processing technology of mechanical parts: Yu et al. [Contribution 4] focused on the repair of surface cracks in mechanical components by combining thermo-mechanical finite element simulations with laser cladding experiments. Repair parameters were optimized, and the mechanical and wear properties of the repaired layers were evaluated. The results showed that the cladding layers produced under optimized conditions outperformed the substrate material in both tensile strength and wear resistance, indicating the strong potential of laser cladding in remanufacturing and surface enhancement of mechanical components. This study further demonstrates that post-processing and repair technologies are not only important complements to manufacturing workflows, but also play a crucial role in extending service life and restoring the performance of key components.
3. Functional/graded materials using advanced manufacturing methodologies: Zeng et al. [Contribution 5] fabricated a silicon nanowire array biosensor for the combined detection of colorectal cancer biomarkers, achieving highly sensitive and label-free detection of ctDNA and CEA, thus demonstrating the potential of micro/nano-manufacturing technologies in biosensing and early disease screening. Chen et al. [Contribution 6] employed electro-spark machining to construct MoCuOx bimetallic oxide electrodes for micro-supercapacitors. The resulting devices exhibited high areal capacitance and excellent cycling stability, indicating the unique advantages of this method for fabricating high-performance micro-scale energy-storage materials. Meanwhile, Ameen et al. [Contribution 7] prepared micro-pillars using focused ion beam techniques and conducted micro-compression experiments to investigate the influence of different phase regions and α/β interfaces on the strength of Ti-6Al-4V alloys. Their work revealed, at the micro-scale, the important role of phase interfaces in plastic deformation and strengthening, thereby providing an experimental basis for microstructural regulation of advanced structural materials. Collectively, these studies show that advanced manufacturing methodologies are not limited to shaping processes themselves, but are increasingly becoming key enablers for the controlled fabrication of functional and graded materials.
4. Modeling and numerical analyses in advanced manufacturing processes: Liu et al. [Contribution 8] developed a coupled electro-thermal-fluid multiphysics model for DC arcs and systematically analyzed the sensitivities of temperature fields, pressure fields, and potential distributions to current and shielding gas flow rate. Their study clarified the energy accumulation and arc constriction behavior under the coupling of high current and high gas flow. Wang et al. [Contribution 9] addressed the influence of material rheology and external disturbances on the precision of food 3D printing by proposing a dual closed-loop collaborative control strategy for temperature and pressure, and experimentally demonstrated the effectiveness of this approach in improving forming accuracy and structural stability. Bai et al. [Contribution 10] investigated the static mechanical performance and thermal management capability of lattice structures fabricated by laser powder bed fusion. Their results showed that the introduction of lattice skeletons can improve heat transfer pathways, enhance temperature uniformity, and improve overall heat dissipation performance. These studies further underscore the central role of numerical simulation, process modeling, and experimental validation in advanced manufacturing research.
5. Advanced detection, monitoring and intelligent control for manufacturing: Fang et al. [Contribution 11] proposed a method for calculating insert flank wear width using an on-machine laser tool setter, enabling online condition assessment and life prediction for indexable face milling tools, and thereby improving machining reliability and tool utilization. Chen et al. [Contribution 12] developed a lightweight hotspot detection model integrating SE and ECA mechanisms, which improved hotspot recognition accuracy while reducing model complexity, illustrating the promise of intelligent algorithms in advanced manufacturing inspection. Qing et al. [Contribution 13] addressed the calibration of installation errors in redundant MEMS-IMU systems for measurement while drilling by proposing a 45°-inclined six-position calibration method, significantly enhancing measurement accuracy and system reliability. From online sensing and data-driven recognition to precision calibration, these studies reflect the rapid development of advanced detection and intelligent control technologies in modern manufacturing systems.

Finally, the guest editors would like to express their sincere gratitude to all the authors for their valuable contributions to this Special Issue, which have greatly advanced the dissemination and development of cutting-edge micro- and nano-manufacturing technologies. We also extend our thanks to the dedicated reviewers, whose time and effort in reviewing the manuscripts have been essential in enhancing the quality of the submissions. We sincerely invite all researchers and professionals to continue submitting their work to this Special Issue. Your insightful contributions will further enrich the ongoing discussions and progress in this field.
